# Oscillatory dynamics underlying noun and verb production in highly proficient bilinguals

**DOI:** 10.1038/s41598-021-04737-z

**Published:** 2022-01-14

**Authors:** Shuang Geng, Nicola Molinaro, Polina Timofeeva, Ileana Quiñones, Manuel Carreiras, Lucia Amoruso

**Affiliations:** 1 Basque Center on Cognition, Brain and Language (BCBL), 20009 San Sebastian, Spain; 2 IKERBASQUE, Basque Foundation for Science, 48009 Bilbao, Spain; 3 University of the Basque Country, UPV/EHU, 48940 Bilbao, Spain

**Keywords:** Neuroscience, Cognitive neuroscience, Language

## Abstract

Words representing objects (nouns) and words representing actions (verbs) are essential components of speech across languages. While there is evidence regarding the organizational principles governing neural representation of nouns and verbs in monolingual speakers, little is known about how this knowledge is represented in the bilingual brain. To address this gap, we recorded neuromagnetic signals while highly proficient Spanish–Basque bilinguals performed a picture-naming task and tracked the brain oscillatory dynamics underlying this process. We found theta (4–8 Hz) power increases and alpha–beta (8–25 Hz) power decreases irrespectively of the category and language at use in a time window classically associated to the controlled retrieval of lexico-semantic information. When comparing nouns and verbs within each language, we found theta power increases for verbs as compared to nouns in bilateral visual cortices and cognitive control areas including the left SMA and right middle temporal gyrus. In addition, stronger alpha–beta power decreases were observed for nouns as compared to verbs in visual cortices and semantic-related regions such as the left anterior temporal lobe and right premotor cortex. No differences were observed between categories across languages. Overall, our results suggest that noun and verb processing recruit partially different networks during speech production but that these category-based representations are similarly processed in the bilingual brain.

## Introduction

Speech production constitutes the bedrock of human communication. This seemingly effortless ability actually depends on a set of complex neural processes, including the retrieval of lexical-semantic information from long-term memory, its translation to articulatory motor programs and the monitoring of what is being verbally expressed^1^.

In daily life conversational settings, nouns and verbs constitute basic components of speech across almost all languages^2^. Nouns and verbs have distinct communicative roles, with the former ones prototypically involving reference to objects, and the latter ones the predication of actions, events and states of being^3^. A growing body of evidence suggests that noun and verb processing are represented in partially non-overlapping networks supporting grammatical and/or lexical/semantic language dimensions, for a review see^4,5^.

Much of what is known about noun and verb representation can be traced back to neuropsychological studies in aphasic patients showing selective difficulties in producing either nouns or verbs after damage to left temporal areas and to fronto-parietal regions, respectively^6–8^. More recently, neuroimaging studies have shown similar patterns of noun–verb dissociation in temporal and frontal regions^9–11^. Moreover, studies using cortical stimulation during awake brain surgery also converge in underscoring a category-based segregation, with greater number of errors in noun naming when stimulating regions in the inferotemporal cortex and greater impairment in verb naming when disrupting activity in prefrontal and parietal areas^12–15^.

At the neurophysiological level, M/EEG studies^16–18^, have reported event-related (i.e., ERP/ERF) differences between nouns and verbs in the P200 and the N400 components which are typically related to lexical access and semantic processing, respectively^19^. For instance, more positive P200 responses in fronto-central motor regions have been found for verbs^16,17^, potentially suggesting that neural generators outside classical language areas may contribute to differences between nouns and verbs. In the case of the N400, a similar effect has been shown, with verbs being overall more positive than nouns^18^. Since this latter component reflects semantic processing, this finding has been interpreted in terms of how meaning-related information from different concepts is retrieved (e.g., visual vs. motoric semantic features), resulting in amplitude and/or topographic differences within this time window.

Nonetheless, these studies have examined the organizational principles governing the neural representation of noun and verb categories in monolingual speakers, leaving unanswered the question of whether a similar organization also stands for bilingual ones. A few recent neuroimaging studies on bilingual speakers have shown similar responses for both languages during noun and verb generation in temporal, parietal, premotor and middle-frontal areas^20,21^, thus supporting the existence of convergent neural substrates across different languages. Yet, there is also evidence^22^ showing that high proficient bilinguals exhibit differential neural patterns of activation for nouns and verbs in their two languages, suggesting that the early bilingual brain can be also sensitive to language-specific properties or, alternatively, that it can be modulated by experiential factors (e.g., proficiency, age of acquisition), even when the L2 is mastered in a native-like fashion.

In this context, inconsistencies yielded by fMRI studies can be potentially overcome with more fine-grained techniques. It could be, for instance, that differences and/or similarities across languages during noun and verb production may rely on temporal and spectral properties of brain activity, which are not captured by fMRI signals. Indeed, hemodynamic responses are slow (i.e., starting approximately ~ 2 s after stimulus presentation) and linguistic functions, occurring on the subsecond time-scale, need to be examined with high-temporal resolution techniques capable of tracking language processing in real-time. Furthermore, fMRI does not allow decomposing brain signals into their different oscillatory rhythms. Interestingly, neurophysiological techniques such as magnetoencephalography (MEG), offer the unique opportunity of capturing this information and, thus to test whether oscillatory dynamics (i.e., the unfolding in time of rhythmic fluctuations) are similar (or not) across languages in the bilingual brain.

It has been suggested that oscillations play a key role in neural communication supporting cognition^23^, providing spectral fingerprints of distinct cognitive operations that would remain blind to traditional evoked analysis (e.g., ERP/ERF), in which responses are phase-locked to the experimental stimulus^24,25^. Indeed, some studies have reported weak spatio-temporal overlap between evoked and rhythmic responses during picture naming, potentially suggesting that the neural processes captured by these two approaches actually differ^26^.

When considering previous M/EEG studies measuring oscillatory dynamics during speech production in monolinguals, theta (4–8 Hz) power increases^27^ and alpha–beta (8–25 Hz) power decreases^28–30^ have been reported in association to the retrieval of lexical-semantic information from long-term memory. In addition, frontal theta power increases during speech production have been related to executive control in the face of increased cognitive demands^31^.

In a recent study^32^ conducted in L1 Spanish speakers, we have shown a different engagement of ventral and dorsal streams during the production of nouns and verbs, respectively; involving decreases in alpha and beta frequency bands between 200 and 500 ms after picture presentation. Yet, whether these oscillatory brain responses remain similar when accessing different categories (i.e., nouns vs. verbs) in bilingual speakers remains largely unexplored.

Here, we sought to move further by shedding light on the neurophysiological signatures of nouns and verbs in two languages within a study population of highly proficient Spanish–Basque bilinguals by means of MEG. Participants were asked to overtly name pictures depicting objects or actions in the context of minimal sentences, thus forcing them to produce utterances involving nouns or action verbs, respectively. Importantly, items from both languages and categories were carefully matched for several variables, including word frequency, familiarity and length^33^.

Overall, under the hypothesis that nouns and verbs are underpinned by different oscillatory brain responses^32^, we predicted distinct alpha–beta patterns for the use of these categories in a time-window typically associated to lexico-semantic processing (~ 200–500 ms). In addition, we expected increased theta power for verbs than nouns likely related to greater processing demands during semantic integration^34^. Indeed, verbs exhibit more shallow relations to other words in the lexicon as compared to nouns, which stay relatively consistent in their meaning^5,35^. Furthermore, based on previous neuroimaging evidence^20,21^ showing that the same neural structures are involved in the differential processing of nouns and verbs in two languages (language invariance) during speech production, we likely expected similar activation patterns in Spanish and Basque potentially reflecting the engagement of similar brain networks across languages.

## Results

### Performance in picture naming

Overall, information from both categories and languages was retrieved equally well (Spanish: ~ 99% for nouns and ~ 98.7% for verbs; Basque: ~ 98.9% for nouns and ~ 98.5% for verbs). Differences in terms of categories emerged when considering reaction time values (RT). More specifically, the RM-ANOVA conducted on them yielded a main effect of category (*F*_1,15_ = 74.61, *p* < 0.0001; partial eta = 0.83) with faster RTs for nouns (Spanish: mean = 1001.47; SD = 454.94; Basque: mean = 961.11; SD = 431.68) as compared to verbs (Spanish: mean = 1115.79; SD = 446.1; Basque: mean = 1133.83; SD = 433.7) independently of the language used to name. No main effect of language (*p* = 0.68) or interaction between category and language (*p* = 0.22) were observed.

### Oscillatory dynamics underlying noun and verb naming in Spanish and Basque

**Figure 1:**
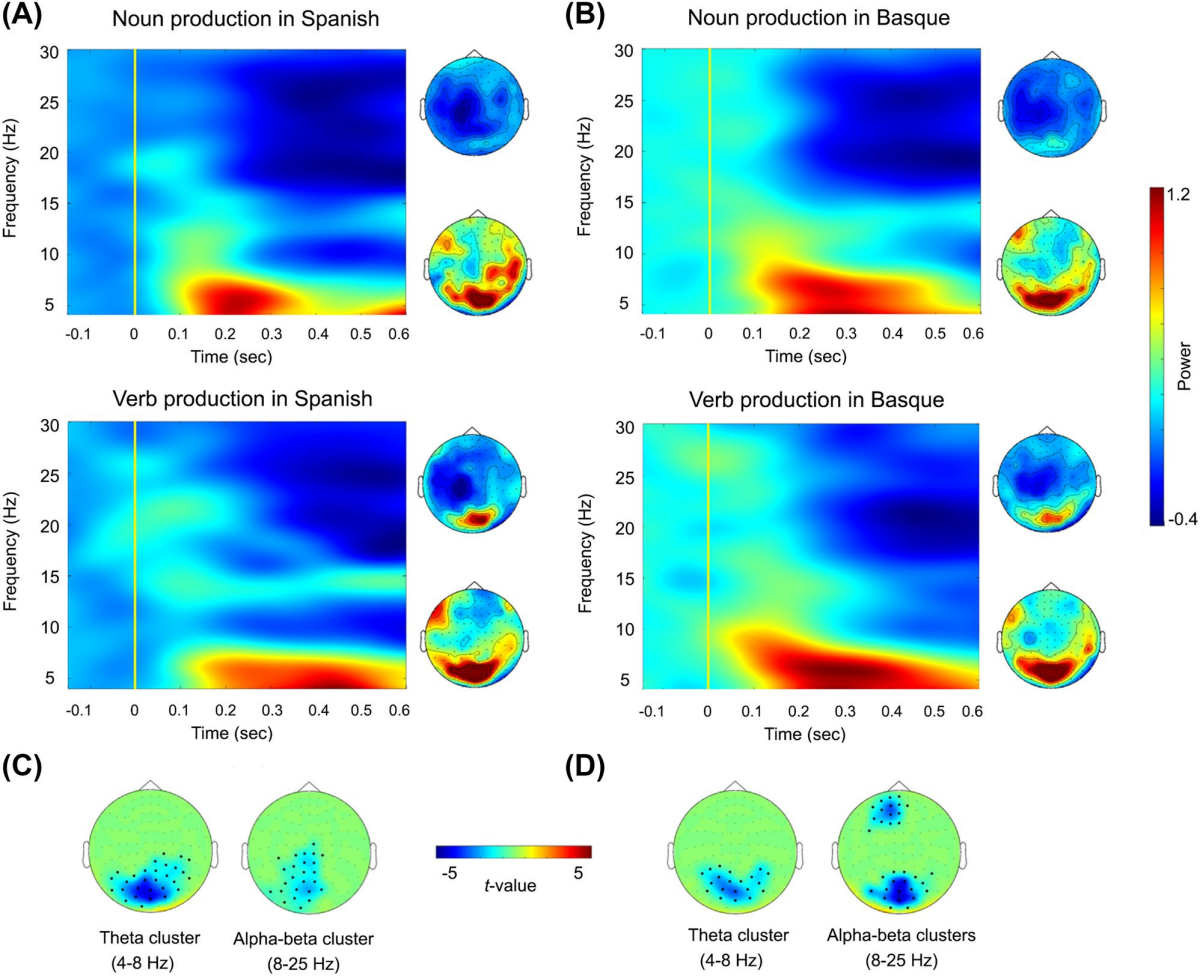
Oscillatory correlates of noun and verb production in bilingual speakers. Time–frequency representations (TFRs) for nouns and verbs in Spanish (**A**) and Basque (**B**). TFRs and topographic distributions plots showing theta and alpha–beta effects are plotted as relative power change compared to the baseline period (500 ms pre-stimulus) in the combined gradiometers highlighted by the significant clusters (**C**, **D**).

As shown by the TFRs depicted in Fig. 1, noun and verb naming in either Spanish or Basque showed theta power increases (4–8 Hz) and alpha–beta (8–25 Hz) power decreases during speech production within the first 500 ms after picture onset.

When comparing noun and verb naming conditions in Spanish, a significant negative cluster was observed in the theta band (4–8 Hz; Monte Carlo *p* = 0.002, two-tailed), with nouns exhibiting less power than verbs. The cluster extended from 100 to 500 ms and comprised bilateral posterior sensors. A significant negative cluster was also observed in the alpha–beta bands (8–25 Hz; Monte Carlo *p* = 0.02, two-tailed), with overall power decreases for nouns as compared to verbs. This cluster was evident from 220 to 500 ms in posterior bilateral sensors as well (See Fig. 1A).

Similarly, when contrasting both categories in Basque, a significant negative cluster (4–8 Hz; Monte Carlo *p* = 0.01, two-tailed), was found in the theta band. Paralleling Spanish findings, nouns exhibited overall less power than verbs in bilateral posterior sensors, as highlighted by a significant cluster extending from 280 to 500 ms. Finally, two negative clusters were found in the alpha–beta range (8–25 Hz; Monte Carlo *p* = 0.01 and *p* = 0.03, two-tailed, respectively; see Fig. 1B), showing decreased power for noun as compared to verb naming. The clusters were evident from 100 to 460 ms and from 180 to 500 ms, with the former comprising posterior sensors and the latter, left frontal ones.

Finally, no significant clusters were observed (all *p*s > 0.2) when comparing noun and verb naming conditions across languages (i.e., Spanish noun vs. Basque noun and Spanish verb vs. Basque verb), in the theta (4–8 Hz) or the alpha–beta (8–25 Hz) frequency bands.

### Source level analysis of category-related effects

**Figure 2:**
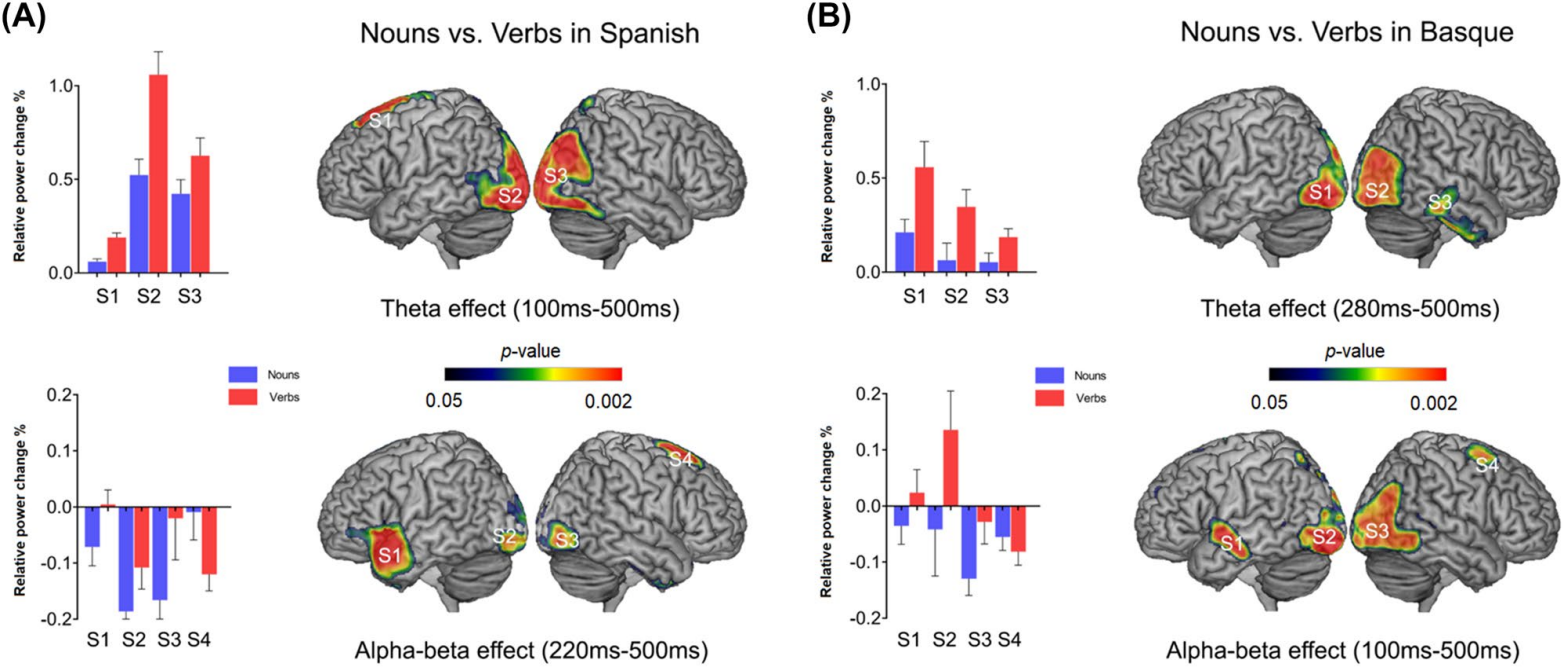
Neural correlates of the TFR sensor-level effects. We localized regions of local maxima with respect to baseline in Spanish (**A**) and Basque (**B**), and restricted between-condition comparisons (nouns vs. verbs) to those sites. Localization of activation peaks was circumscribed to the theta (4–8 Hz) and alpha–beta (8–25 Hz) frequency bands in the time intervals highlighted by the significant clusters in each language. For visualization purposes, we use bar plots showing relative power change for each category (nouns in blue and verbs in red) at each peak maxima to clarify the direction of the effect. All plotted regions reached a *p* value < 0.05.

Significant oscillatory effects at the sensor level were source reconstructed considering the frequency-bands and time-windows highlighted by the significant clusters. In the case of Spanish (see Fig. 2A), theta peaks were found bilaterally in visual cortices and in the left SMA. For Basque (see Fig. 2B), these peaks were localized in bilateral visual cortices as well and in the right middle temporal gyrus. In all cases, regions showed increased power for verbs as compared to nouns.

Brain regions likely contributing to the alpha–beta effects in Spanish, on the other hand, were found in bilateral visual cortices, the left anterior temporal lobe and the right premotor area. While the former regions showed more desynchronization for nouns as compared to verbs, the latter one, namely premotor, showed a reversed pattern, with more desynchronization for verbs as compared to nouns. In the case of Basque, the involved regions were the bilateral visual cortices, the left superior anterior temporal lobe and the right premotor area, with occipito and temporal regions showing stronger alpha–beta power decreases for nouns as compared to verbs, and the right premotor area showing power decreases for verbs as compared to nouns.

## Discussion

In the present study, we sought to investigate the spectro-temporal and neural underpinnings of noun and verb production in highly proficient Spanish–Basque bilinguals. To this end, MEG signals were recorded while participants performed a picture naming task, the gold standard for studying the cognitive architecture of speech production^36^. Overall, bilingual speakers showed similar oscillatory patterns within the first 500 ms after picture onset, exhibiting theta power increases and alpha–beta power decreases regardless of the condition and the language used to name. When comparing noun and verb conditions separately in Spanish and Basque, stronger theta power increases in the case of verbs and stronger alpha–beta power decreases in the case of nouns, were observed irrespectively of the language at use. Regions involved in the theta modulations were localized in occipital cortices, MTG and SMA, showing in all cases increased power for verbs than nouns. Candidate regions mostly contributing to the alpha–beta scalp effects were localized in occipital and temporal regions in the case of nouns and in premotor cortices in the case of verbs. When comparing noun and verb conditions across languages no differences were observed in any of the frequency-bands of interest. Of note, these results were mirrored at the behavioural level, with RTs showing no differences in noun and verb processing across languages. All in all, our findings underscore the existence of common oscillatory dynamics in Spanish and Basque, suggesting that the core principles governing the organization of lexico-semantic representations and their retrieval in the bilingual brain are similar in both languages, at least when considering highly proficient bilinguals.

A large body of neuroimaging evidence suggests that multiple languages share a common neuroanatomical system, with differences in L1 and L2 reflecting varying computational demands mediated by factors such as proficiency, age of acquisition, and level of language exposure^37,38^. In this context, the presence of noun–verb dissociations sustained in two languages in highly proficient bilinguals has been previously reported in fMRI^20,21,39^ and brain lesion studies^40,41^. These findings have been taken as evidence for the existence of language-invariant cortical mechanisms in bilingual speakers while processing lexico-semantic representations during speech production.

Here, we add converging evidence from a neurophysiological standpoint by showing that the oscillatory fingerprints supporting noun–verb dissociations are similar across languages in highly proficient Spanish–Basque bilinguals.

In line with the view that the retrieval of lexico-semantic information is enabled via power decreases of alpha–beta (8–25 Hz) oscillations^31^, we observed reduced alpha–beta power for nouns as compared to verbs in both Spanish and Basque, suggesting that similar mechanisms as those used by monolinguals might be called to play in bilingual speakers when both languages are mastered in a native-like fashion. This is also in keeping with previous evidence from our lab^32^, showing that L1 Spanish speakers recruit different networks in the alpha and beta bands while processing nouns and verbs, with a stronger involvement of occipital and temporal nodes within the ventral stream in the case of nouns; and of premotor and superior parietal nodes within the dorsal stream in the case of verbs, at least when underscoring semantic aspects dissociating these categories (i.e., objects vs. actions). More specifically, noun and verb representations are known to differ in terms of feature types, with visual features more represented in the object domain and sensory-motor features in the action one^42,43^. This view predicts that, at a neural level, the lexical retrieval of object nouns will mainly recruit occipito-temporal regions storing visual features, while the retrieval of action verbs will mainly recruit motor/premotor regions storing sensory-motor features^44–46^. In line with this view, we observed that premotor areas, were more strongly involved in verb processing as shown by stronger alpha–beta power decreases in either Spanish or Basque. Furthermore, visual areas in bilateral occipital cortices and the anterior temporal lobe (ATL) were highlighted by our source analysis in both languages as being more involved in noun naming. The engagement of bilateral visual areas was well expected given the nature of the task, which implies the recognition of the item to be named as a necessary stage occurring within ~ 200 ms of picture presentation^47–49^. The ATL, on the other hand, has been proposed as a hub region in semantic processing^50,51^ and there is evidence supporting its involvement during object recognition and overt naming^52–55^. Furthermore, it has been shown that this region houses language-invariant semantic representations in bilinguals^56,57^.

Lexico-semantic processing, however, does not occur in isolation but rather is closely intertwined with cognitive control during speech production. In this regard, the finding of higher theta power for verbs as compared to nouns seems to reflect the increased semantic processing demands imposed by the different categories. This finding is in keeping with previous evidence^34^ showing similar theta power increases for verbs as compared to nouns in monolinguals. In that study, the authors interpreted theta power modulations in terms of differences in the semantic organization of noun and verb categories. Indeed, while concrete nouns are known to share many semantic features among different levels; verbs are more abstract and typically exhibit a shallower semantic organization^5^. Cortical MEG peaks contributing to the theta effects were found in bilateral visual cortices for both languages, in the right middle temporal gyrus (MTG) for Basque and in the left SMA for Spanish, in all cases showing increased power for verbs as compared to nouns. Of note, the right MTG and left SMA have been highlighted as important nodes in the inhibition network and also reported to be involved in bilingual management of two languages in functional neuroimaging studies^58^. Furthermore, neurophysiological evidence^59^ suggests that theta increases in the SMA and posterior visual cortices—starting around ~ 150 ms and continuing throughout the task—may index initiation of the item search in memory, reflecting the retrieval of semantic features with different levels of complexity. Nevertheless, it is also true that even though pictures denoting nouns and verbs were carefully matched for many linguistic variables, the visual complexity of pictures depicting actions might have been higher than the one exhibited by single objects. Since we did not measure this aspect (e.g., by asking participants to rate the pictures), we cannot completely rule out that theta power increases for verbs as compared to nouns, sourced in visual cortices, could actually reflect task-related visual attention differences rather than top-down control mechanisms. Indeed, visual theta rhythms have been previously linked to sustained attention^60–62^. This aspect needs to be addressed by future studies experimentally dissociating visual and linguistic stages during speech production or, alternatively, by using the same pictorial stimuli for the different categories.

Another aspect that needs to be considered is that, at the source level, MEG peaks contributing to maximal differences between noun and verb naming did not completely overlap for Spanish and Basque. Specifically, while both languages showed comparable activations in bilateral visual cortices, left anterior temporal areas and right premotor cortex; regions involved in the theta effect (i.e., SMA and MTG) differed. This might be explained by timing and/or methodological aspects, potentially reflecting that within the first 500 ms after stimulus onset, brain regions in the language network may have differently contributed, in terms of power engagement, to the category-related effects in the different languages. Furthermore, the timing of the significant clusters involved in the theta and alpha–beta effects varied across languages. Specifically, the one found in Spanish within the theta frequency-band started earlier in time as compared to the one observed in Basque (i.e., 100 ms and 280 ms after stimulus onset, respectively). While the timing of the cluster is not indicative of the onset of the effect^63^, the source localization was performed on the significant time-windows highlighted by them. Thus, it is likely that this methodological aspect may have also played a role. Alternatively, observed differences may stem from the fact that, despite the balanced mastery of both languages, Basque was acquired in most cases as the L2. Although L2 was acquired early, neurophysiological specialization may nevertheless differ between the languages and this might explain that areas supporting noun–verb segregation do not entirely converge, even though they are recruited via similar oscillatory mechanisms.

Nevertheless, MEG source localization has its own limitations given the ill-posed inverse problem, and fine-grained statements about underlying cortical sources cannot be formulated with the approach used in the present study. Further research zooming into these aspects are required to disentangle this issue.

Another aspect that requires further consideration relates to the overt nature of the task. Indeed, as highlighted by^64^, speech production tasks have been long avoided in neurophysiological studies due to the potential existence of muscle artifacts, which may lead to a bad signal-to-noise ratio in the recordings. Here, we focused on the first 500 ms after picture onset—which can be considered as a “safe” window of artifact-free brain responses^65^—and ran state-of-the-art pipelines for semiautomatic detection of muscle artifacts^66^. Yet, even if we only considered the initial 500 ms time-window for the final analysis, it is true that these methods reflect the deviation of the whole epoch (i.e., 1000 ms in our case) and look for abnormality given the evolution of the recording. Thus, we cannot completely rule out that the segments of analysis may have still contained some noise. Nevertheless, we find this unlikely for several reasons. First, previous studies have used a similar methodological approach leading in all cases to adequate estimates of brain non-contaminated activity^30,32,67,68^. Second, the beamformer technique used here to reconstruct underlying brain sources is known to attenuate myogenic artifacts by suppressing signals whose spatial scalp distribution cannot be explained by a dipolar source in the brain (please see^30^ for a further discussion of this aspect). Third, the specificity of the observed effect (i.e., increased power for verbs in premotor structures as compared to nouns) speaks in favor of a category related modulation rather than the presence of myogenic activity and fits well with evidence showing that motor alpha–beta oscillations play a key role in action semantics^69–71^.

Finally, it is worth mentioning that our sample size was rather small, although not smaller than those reported in similar MEG studies approaching the study of speech production in bilinguals^72–76^. Thus, future studies with larger sample sizes are needed to strengthen our conclusions.

## Conclusions

Overall, in the present study, we show that the oscillatory networks involved in noun and verb production in highly proficient bilinguals exhibit similar theta (4–8 Hz) and alpha–beta (8–25 Hz) dynamics across languages. Specifically, the finding of theta power increases for verbs and alpha–beta power decreases for nouns irrespectively of the language at use underscores the existence of common principles supporting the organization and retrieval of lexico-semantic information in bilingual speakers, at least during early stages of speech production. While similar modulations in low-frequency brain rhythms have been previously reported in monolingual speakers, to the best of our knowledge, this is the first study in showing that comparable oscillatory patterns also stand for highly proficient bilinguals.

## Methods

### Participants

A total of 20 Spanish–Basque bilinguals were recruited through the BCBL database and received economical compensation for their participation in the study. However, four participants were discarded from the study due to excessive artifacts in MEG recordings. Thus, all subsequent behavioural and MEG statistical comparisons were performed on a total of 16 participants (4 male, M = 25.87; SD = 5.25). All participants but one reported Spanish as the first language (L1) and Basque as the second one (L2). Language proficiency was assessed with the Basque, English, and Spanish Test [BEST]^77^, using the semi-structured interview part of the test which measures fluency, lexical resources, grammatical constructions and pronunciation (Likert-like scale with scores ranging from 1 to 5). The cut-off criteria for considering an individual as a high-proficient bilingual were scores ≥ 4 in their L2. The nonparametric Wilcoxon signed rank test showed no significant differences (*p* = 0.053) between Spanish (M = 5; SD = 0) and Basque (M = 4.83; SD = 0.33), indicating that participants had comparable proficiency in both languages. In addition, no significant differences (*t* = − 0.62, *p* = 0.53) were observed in the age of acquisition (AoA) between Spanish (M = 0.62; SD = 1.2; range: 0–3 years) and Basque (M = 0.93; SD = 1.12; range: 0–3 years), with both languages being acquired early in life. All participants were right-handed as measured by the Edinburgh Handedness Inventory^78^, possessed normal or corrected-to-normal vision and no history of neurological or psychiatric disease. The Ethics and Scientific Committee of the BCBL, following the declaration of Helsinki, approved the study protocol. All participants gave their written informed consent prior to the study.

### Stimuli and task

Language production was assessed using the MULTIMAP, a multilingual picture naming task for mapping the language network developed by our group^33^. MULTIMAP consists of an open-access database of standardized color pictures representing objects and actions. These pictures have been tested for name agreement with speakers of different languages including Spanish and Basque, and have been controlled for relevant linguistic features (e.g., word frequency, word length, number of letters, number of phonemes, number of syllables, number of substitution neighbors, familiarity, imageability, and concreteness) in cross-language combinations.

In separate blocks, participants were instructed to observe the pictures and name them overtly in Spanish or Basque as quickly and accurately as possible. Production of nouns and verbs was requested in the context of short sentences, which is a more ecological form of speech than isolated naming. More specifically, on top of the object-related images we added the text “Esto es…” or “Hori da” [“This is…” in Spanish and Basque, respectively] to force the production of a short sentence that had to agree in number with the target noun (e.g. “Esto es una manzana” in Spanish or “Hori da sagarra” in Basque, English translation: “This is an apple”).

Similarly, on top of the action-related pictures, we included “El…”/“Ella…” or “Hark…” [“He…” or “She…” in Spanish and Basque]. This introductory text was used as a cue for the production of a sentence that started with the given subject and had a finite verb form in 3rd person singular (e.g. Spanish: “Él corta”, Basque: “Hark ebakitzen”, English translation: “He cuts”).

We used MATLAB Release 2012b (The MathWorks, Inc., Natick, Massachusetts, United States) and Cogent Toolbox for picture presentation. Trials started with a fixation cross lasting for 1000 ms, followed by the stimulus displayed for 2 s. ISI randomly varied between 3 and 4 s. A total of 80 picture items (i.e., 40 for nouns and 40 for verbs) were used. Each picture was presented twice for a total of 80 trials per condition. Each block lasted ~ 10 min, and participants were allowed to take a short break between them.

### MEG and MRI recordings

Neuromagnetic signals were continuously recorded by means of an Elekta Neuromag 306-channels system (Helsinki, Finland) in a shielded room at a sampling rate of 1000 Hz. MEG signals were online filtered with a passband between 0.1 and 330 Hz and sampled at 1 kHz. Participant's head position inside the helmet was monitored with five head position indicator coils (HPI) located on the scalp, throughout the experiment. Six electrode pairs were used to control for ocular (i.e., placed in the external chanti of each eye and above and below the right eye) and cardiac activity (i.e., placed below the right clavicle and under the left rib bone). Three anatomical fiducials (i.e., nasion and left and right prearicular points) plus ~ 300 additional points registered over the scalp and nose area were digitalized and further used to spatially align the MEG sensor coordinates to the native T1 high-resolution 3D structural MRI of each participant. Structural MRIs were acquired with a Siemens 3 T magnetom prismafit MR scanner (Siemens, Munich, Germany) in a separate session using the following parameters: echo time = 2.97 ms, repetition time = 2530 ms, flip angle = 7° and field of view = 256 × 256 × 176 mm^3^, number of axial slices = 176, slice thickness = 1 mm, in-plane resolution = 1 mm × 1 mm.

### Behavioural assessment

Participant’s vocal responses were recorded and monitored online by a research assistant during the task. Automatic detection of naming latencies was done with the Chronset tool^79^. Erroneous responses or utterances containing disfluencies were excluded from the final analyses. Response latencies were trimmed at 2.5 standard deviations (SD) above participant’s mean in each condition and analysed using a 2-way ANOVA with Language (Spanish, Basque) and Category (Noun, Verb) as within-subject factors.

### Data preprocessing

Continuous MEG data were initially pre-processed off-line using the temporal extension of the signal space separation method^80^ implemented in Maxfilter 2.2 (Elekta-Neuromag), which allows for external magnetic noise suppression, head movement correction and bad channels interpolation. MEG analyses were performed using FieldTrip (version 20170911)^66^ in MATLAB Release 2014b. Data were down-sampled to 500 Hz and segmented into epochs from 500 ms before picture onset to 1000 ms after picture onset.

A semi-automatic procedure was then employed to remove epochs with myogenic activity, SQUID jumps and flat signal related artefacts. To this end, we used the Fieldtrip function ft_artifact_zvalue. This algorithm computes a z-score time-course for each sensor by subtracting the mean and dividing by the standard deviation across trials. The obtained z-values are then averaged across sensors providing an index of the global standardized deviation. Afterward, a threshold for the global z-score is chosen in order to reject those epochs deviating from it. Muscle artifacts and SQUID jumps were independently identified with different sets of parameters (e.g., sensors to consider, filtering bands, type of padding). In all cases, the default Fieldtrip parameter values were used. Finally, a fast independent component analysis (ICA) was used to correct for heartbeat and eye movement artefacts. ICA rejection was performed manually based on the topographical patterns of the components. One clear eye-movement component and one heartbeat component were removed for all participants. Importantly, no significant differences were observed (all *p* values > 0.066) between conditions or languages in terms of the number of trials kept for the final MEG analysis (Spanish: nouns *M* = 55.94, *SD* = 8.96; verbs, *M* = 52.88, *SD* = 8.19; Basque: nouns, *M* = 53.75, *SD* = 7.21; verbs, *M* = 53.94, *SD* = 7.87).

### Data analysis

Time–frequency representations (TFR) were obtained for clean MEG data segments in the theta (4–8 Hz) and the alpha–beta (8–25 Hz) frequency-bands using Hanning tapers and a fixed window length of 500 ms advancing in 10 ms steps. These frequency-bands were selected based on previous M/EEG literature suggesting a role for theta in cognitive control during semantic integration^34^ and a role for alpha–beta oscillations^28–30^ in the retrieval of lexical-semantic information from long-term memory.

Power estimates were calculated separately for each orthogonal direction of a gradiometer pair and then combined, resulting in a total of 102 measurement sensors. Power was expressed as relative change with respect to a ~ 500 ms pre-stimulus baseline period.

Cluster-based permutation tests^81^ were used to assess power differences between languages and categories at the sensor-level. For the contrasts, we averaged over frequency bins of interest (i.e., 4–8 Hz and 8–25 Hz) while considering all time-points between 0 and 500 ms after picture onset and all 102 combined gradiometers, since no a-priori hypotheses about timing or locations were held. The 0–500 ms time-window was chosen based on previous neurophysiological evidence^64^ suggesting that, in overt speech production tasks, artifact-free recordings (e.g., not contaminated with articulatory activity) can be safely acquired during this period.

The permutation *p* value was calculated using the Monte-Carlo method with 1000 random permutations. The alpha threshold for significance testing was a *p*-value below 5% (two-tailed).

### MEG source reconstruction

Source reconstruction was performed in order to estimate the brain regions likely contributing to the sensor-level effects. Anatomical MRI data from each participant (T1-weighted) was segmented using the Freesurfer software^82^. Co-registration between MEG sensor and individual’s MRI coordinates was manually performed by aligning the digitized head-surface and the three fiducial points to the outer scalp surface. The forward model was calculated using the Boundary Element Method (BEM) implemented in MNE suite (RRID:SCR_005972,^83^ for three orthogonal tangential current dipoles, placed on a homogeneous 5-mm grid covering the entire brain. For each source, the forward model was then reduced to its two principal components of highest power, which closely correspond to sources tangential to the skull. All sensors (i.e., planar gradiometers and magnetometers) were used in the source localization analysis. Each sensor signal (and the corresponding forward-model coefficient) was normalized by its noise variance (estimated from the 500 ms baseline period prior to picture onset).

Based on previous studies from our lab^32^, we used the Linearly Constrained Minimum Variance (LCMV) method for estimating brain source activity^84^. Cross-spectral density (CSD) matrices were calculated in the time–frequency window of the significant sensor-level effects and in an equally-sized baseline period. The real part of the combined matrices was used to compute a common filter (i.e., LCMVB beamformer).

In order to run group-level analysis, brain maps were transformed from the individual MRIs to the standard MNI using the spatial-normalization algorithm implemented in Statistical Parametric Mapping (SPM8, Wellcome Department of Cognitive Neurology, London, UK).

Then we identified the coordinates of the local maxima in group-level power maps with respect to baseline and restricted statistical comparisons between conditions to those sites. Local maxima were defined as contiguous voxels displaying higher power than all other neighboring voxels^85^. Group-level difference maps were calculated by subtracting *f*-transformed trial and baseline group-level power maps for each frequency of interest. Under the null hypothesis that power maps are the same regardless the experimental condition, genuine and baseline levels are exchangeable at the participants-level prior to difference map computation. In order to reject this hypothesis and compute a statistical significance threshold for the correctly labelled difference map, the sample distribution of the maximum of the difference map’s absolute value was computed using a permutation approach. The threshold at *p* < 0.05 was estimated as the 95 percentile of the sample distribution. All supra-threshold MEG peaks were interpreted as indicative of brain regions likely contributing to the sensor-level effects.

The coordinates of significant local power maxima were statistically compared using the location-comparison method^86^. This robust method uses a bootstrap approach^87^ to build a permutation distribution of the coordinates of the local maxima in two conditions and tests the probability that the distance between them is zero^88^. To do so, it uses a multivariate location test similar to the Hotelling T^2^ test, which is the multivariate extension of the classical Student t-test. Importantly, the location-comparison method has shown to successfully deal with spectral leakage problems resulting from directly contrasting brain maps from different conditions.

## Data Availability

All the data that support the findings of this study as well as the code for data preprocessing and analysis are available on request from the corresponding author.
