# Author Correction: Oscillatory dynamics underlying noun and verb production in highly proficient bilinguals

**DOI:** 10.1038/s41598-023-30136-7

**Published:** 2023-02-20

**Authors:** Shuang Geng, Nicola Molinaro, Polina Timofeeva, Ileana Quiñones, Manuel Carreiras, Lucia Amoruso

**Affiliations:** 1 Basque Center On Cognition, Brain and Language (BCBL), 20009 San Sebastian, Spain; 2 IKERBASQUE, Basque Foundation for Science, 48009 Bilbao, Spain; 3 University of the Basque Country, UPV/EHU, 48940 Bilbao, Spain

Correction to: *Scientific Reports* 10.1038/s41598-021-04737-z, published online 14 January 2022

The Acknowledgements section in the original version of this Article was incomplete.

“This research was supported by the Basque Government through the BERC 2022-2025 program and by the Spanish State Research Agency through BCBL Severo Ochoa excellence accreditation CEX2020-001010-S, by the Ikerbasque Foundation, by a Juan de la Cierva Fellowship to LA (IJCI-2017-31373) and by the Plan Nacional RTI2018-096216-A-I00 (MEGLIOMA) to LA and RTI2018-093547-B-I00 (LangConn) to MC and IQ both funded by the Spanish Ministry of Economy and Competitiveness.”

now reads:

“This research was supported by the Basque Government through the BERC 2022-2025 program and by the Spanish State Research Agency through BCBL Severo Ochoa excellence accreditation CEX2020-001010-S, by the Ikerbasque Foundation, by a Juan de la Cierva Fellowship to LA (IJCI-2017-31373) and by the Plan Nacional RTI2018-096216-A-I00 (MEGLIOMA) to LA and RTI2018-093547-B-I00 (LangConn) to MC and IQ both funded by the Spanish Ministry of Economy and Competitiveness. This research was also supported by the Fundación Científica AECC (FCAECC) through the project PROYE20005CARR".

The original Article has been corrected.

